# 基于超高效液相色谱-线性离子阱-静电场轨道阱质谱、网络药理学和动物实验探究川西合耳菊治疗急性湿疹的作用机制

**DOI:** 10.3724/SP.J.1123.2025.10013

**Published:** 2026-07-08

**Authors:** Cizhen DANZENG, Xinchen FU, Paier LUO, Yachun LUO, Meijuan SHAO, Shuya XIE, Haoyu GE, Xifan PENG, Zhaxi PUBU, Zhihong YAN

**Affiliations:** 1. 西藏藏医药大学临床教研室，西藏 拉萨 850000; 1. Clinical Teaching and Research Office，Xizang Tibetan Medical University，Lhasa 850000，China; 2. 江西中医药大学药学院，江西 南昌 330004; 2. College of Pharmacy，Jiangxi University of Traditional Chinese Medicine，Nanchang 330004，China; 3. 西藏自治区藏医院皮肤科，西藏 拉萨 850000; 3. Department of Dermatology，Tibetan Hospital of Xizang Autonomous Region，Lhasa 850000，China

**Keywords:** 川西合耳菊, 超高效液相色谱-线性离子阱-静电场轨道阱质谱, 急性湿疹, 网络药理学, 分子对接, *Synotis solidaginea*, ultra performance liquid chromatography-linear trap quadrupole-Orbitrap mass spectrometry （UPLC-LTQ-Orbitrap-MS）, acute eczema, network pharmacology, molecular docking

## Abstract

采用静电场轨道阱质谱（UPLC-LTQ-Orbitrap-MS）法，结合二级质谱图和文献鉴定川西合耳菊化学成分，通过TCMSP数据库和SwissTargetPrediction数据库筛选成分靶点，采用Gene Cards数据库、OMIM数据库筛选急性湿疹的相关靶点，川西合耳菊成分靶点与疾病靶点取交集得到潜在作用靶点，使用String网站及Cytoscape 3.10.1软件构建蛋白质-蛋白质相互作用网络，对潜在靶点进行基因本体论（GO）、京都基因与基因组百科全书（KEGG）分析，运用Cytoscape 3.10.1软件构建成分-靶点-通路网络图，并对关键靶点进行核心化合物分子对接。设计动物实验验证，通过2，4-二硝基氯苯诱导，建立了昆明小鼠（KM小鼠）急性湿疹模型，从整体动物水平评估其疗效，并采用苏木精-伊红染色（HE）、酶联免疫吸附测定、实时荧光定量聚合酶链反应（qRT-PCR）及蛋白质印迹法等技术探讨其抗炎及调控信号通路的作用。结果表明，从川西合耳菊中鉴定出59种化合物，并进一步筛选出7个潜在活性成分：异鼠李素、山柰酚、木犀草素、槲皮素、杨梅素、甜菜碱和迷迭香酸。通过靶点数据库预测，这7个成分共对应192个蛋白质作用靶点。同时，从疾病数据库中检索获得2 214个与急性湿疹相关的蛋白质靶点。将上述两组靶点进行映射（取交集），共获得75个共同靶点，这些蛋白质被认为是川西合耳菊治疗急性湿疹的潜在关键靶点；分子对接结果表明异鼠李素、木犀草素、槲皮素、山柰酚、杨梅素与蛋白质靶点原癌基因酪氨酸蛋白激酶SRC及基质金属蛋白酶9均有较好的结合能力。动物实验表明，川西合耳菊可降低肿瘤坏死因子α（TNF-α）、白细胞介素6（IL-6）以及白细胞介素17（IL-17）的含量，升高干扰素γ的含量来恢复辅助性T细胞1/辅助性T细胞2（Th1/Th2）平衡，通过抑制磷脂酰肌醇3-激酶/蛋白激酶B（PI3K/Akt）信号通路来减轻炎症反应，改善急性湿疹小鼠的临床症状。综上，该方法快速、有效、全面地分析了川西合耳菊中的化学成分，揭示了川西合耳菊治疗急性湿疹的作用机制，为川西合耳菊的进一步开发利用提供了参考依据。

## Abstract (en)

This study employed an integrated strategy combining ultra performance liquid chromatography-linear trap quadrupole-Orbitrap mass spectrometry （UPLC-LTQ-Orbitrap-MS）， network pharmacology， molecular docking simulations， and in vivo animal experimentation to systematically analyze the active components of *Synotis* *solidaginea* （*S. solidaginea*） and to elucidate its underlying therapeutic mechanism in the treatment of acute eczema. Initially， the UPLC-LTQ-Orbitrap-MS method was utilized to perform a comprehensive chemical profiling of *S. solidaginea* extract. Based on accurate mass， multistage fragmentation spectra， and cross-validation with literature and reference standards， 59 constituents were unambiguously or tentatively identified. Subsequently， a network pharmacology framework was established to predict the potential pharmacological mechanisms of the identified compounds. Among the characterized constituents， seven bioactive ingredients—namely isorhamnetin， kaempferol， luteolin， quercetin， myricetin， betaine， and rosmarinic acid—were selected as the primary active components based on their relative abundance and favorable oral bioavailability and drug-likeness properties as predicted by the Traditional Chinese Medicine Systems Pharmacology Database and Analysis Platform （TCMSP）. The corresponding potential protein targets of these seven compounds were systematically screened using the TCMSP database and the SwissTargetPrediction tool， resulting in the identification of 192 component-associated targets after the removal of duplicate entries. Concurrently， a comprehensive collection of disease-associated targets was curated for acute eczema. By querying the GeneCards database and the Online Mendelian Inheritance in Man （OMIM） database with the keyword “acute eczema”， a total of 2 214 distinct therapeutic targets relevant to the pathogenesis and progression of the dermatological condition were retrieved. Through the construction of a Venn diagram to map the component-related targets onto the disease-specific target set， a core network comprising 75 overlapping targets was identified as the potential therapeutic intersection through which *S. solidaginea* exerts its pharmacological effects against acute eczema. To further investigate the functional interactions and biological significance of these 75 intersecting proteins， a protein-protein interaction （PPI） network was constructed using the STRING database （version 12.0） and subsequently visualized and topologically analyzed using Cytoscape 3.10.1 software. Network topological analysis， specifically evaluating the degree centrality， betweenness centrality， and closeness centrality， highlighted SRC proto-oncogene tyrosine-protein kinase and matrix metalloproteinase 9 （MMP9） as the most prominent hub nodes within the network， suggesting their pivotal roles in mediating the therapeutic activity. Furthermore， Gene Ontology （GO） functional annotation and Kyoto Encyclopedia of Genes and Genomes （KEGG） pathway enrichment analyses were conducted on the 75 potential targets. The GO enrichment analysis indicated that these targets were predominantly involved in biological processes related to the regulation of inflammatory responses， cellular response to oxidative stress， and positive modulation of cytokine production. The KEGG pathway analysis further revealed that the therapeutic effects of *S. solidaginea* are likely orchestrated through the modulation of several critical signaling cascades， with the PI3K/Akt signaling pathway being the most significantly enriched pathway. To validate the credibility of these computational predictions at the molecular level， molecular docking simulations were performed to evaluate the binding affinities and interaction modes between the core active flavonoids and the identified hub targets. The docking results demonstrated that isorhamnetin， luteolin， quercetin， kaempferol， and myricetin all exhibited strong and stable binding conformations within the active catalytic pockets of both SRC and MMP9. These compounds were observed to form critical hydrogen bonds and hydrophobic interactions with key amino acid residues， indicating a favorable thermodynamic profile and a high potential for functional inhibition of these key targets in vivo. The final phase of this investigation involved experimental validation using an established murine model of acute eczema. The model was successfully induced on the dorsal skin of KM mice through repeated topical sensitization and challenge with 2，4-dinitrochlorobenzene. Following model establishment， the therapeutic impact of *S. solidaginea* administration was rigorously assessed at the macroscopic， microscopic， and molecular levels. Macroscopic observation and scoring of clinical symptoms revealed a significant amelioration of erythema， edema， and excoriation in the treatment groups. Histopathological examination of skin tissue sections stained with hematoxylin and eosin （HE） confirmed that *S. solidaginea* treatment markedly reduced epidermal hyperplasia， spongiosis， and dermal inflammatory cell infiltration. Mechanistically， quantitative analysis via enzyme-linked immunosorbent assay （ELISA） demonstrated that *S. solidaginea* treatment effectively down-regulated the serum concentrations of the key pro-inflammatory cytokines tumor necrosis factor-alpha （TNF-α）， interleukin-6 （IL-6）， and interleukin-17 （IL-17）， while concurrently restoring the level of interferon-gamma （IFN-γ）. This modulation of cytokine profiles indicates a beneficial shift toward restoring the T helper type 1 cell/T helper type 2 cell （Th1/Th2） immune balance， which is typically dysregulated in acute eczematous conditions. Most importantly， Western blot analysis of the lesional skin tissue provided direct biochemical evidence that the extract of *S. solidaginea* significantly inhibited the phosphorylation of phosphatidylinositol 3-kinase （PI3K） and protein kinase B （Akt）， thereby confirming the suppression of the PI3K/Akt signaling pathway in vivo. In conclusion， this methodological approach enables the rapid， effective， and comprehensive characterization of the phytochemical profile of *S. solidaginea*. Furthermore， this study preliminarily elucidates that the therapeutic mechanism of this medicinal plant in treating acute eczema involves a multi-component and multi-target synergistic action， primarily through the alleviation of the cutaneous inflammatory response and the restoration of immune homeostasis via the inhibition of the PI3K/Akt signaling axis. These findings provide a robust scientific foundation and a valuable reference for the further development， clinical application， and quality control of *S. solidaginea*.

湿疹是一种过敏性的皮肤炎症性疾病，发病机制不明确。急性湿疹是湿疹的早期阶段，有起病急、病程短、瘙痒剧烈等特点，不及时治疗易反复发作^［1］^。目前，湿疹的临床治疗仍面临诸多挑战，包括疾病的复发率高、症状表现异质性强、针对中重度患者的现有药物疗效有限以及缺乏统一且高效的治疗方案等^［1］^。川西合耳菊又称川西尾药菊，为菊科合耳菊属植物川西合耳菊*Synotis* *solidaginea* （*S. solidaginea*）的干燥地上部分^［1］^。该药材主要分布于西藏、四川、云南等地区。《中国植物志》及《藏药志》中记载川西合耳菊具有清热解毒、消炎接骨等功效，能清肝胆诸热，可治疗伤口发炎、肿胀、疮痈、皮炎等疾病^［2-5］^，在西藏地区可用于治疗湿疹。然而，目前尚没有文献对其治疗急性湿疹的作用机制进行研究。

植物的药效主要受其物质基础影响，川西合耳菊化学成分复杂，已报道主要包含黄酮类、酚酸类及生物碱等多种结构类型^［1］^。然而，该类天然药物通常具有成分种类多、含量差异大、结构相似化合物丰富以及存在大量同分异构体等特点，传统的检测方法在成分解析方面存在明显局限：一方面，紫外检测依赖特征吸收峰，难以区分结构相似的化合物^［6］^；另一方面，低分辨质谱在复杂基质中难以实现精确分子式判定，且对于微量成分及同分异构体的区分能力有限^［7］^。因此，建立一种高分辨、高灵敏且具有系统解析能力的分析技术来全面揭示川西合耳菊的化学物质基础，对于深入探究其治疗急性湿疹的作用机制具有重要意义。超高效液相色谱-线性离子阱-静电场轨道阱质谱法（UPLC-LTQ-Orbitrap-MS）通过将Orbitrap高分辨质量分析器与线性离子阱多级质谱（MS*ⁿ*）技术相结合^［8］^，不仅能够提供高达百万分之一质量精度的分子离子信息，实现复杂样品中化合物的精确分子式推断，还能够通过多级碎片信息深入解析化合物的结构特征，从而显著提高天然产物体系中微量成分和结构类似物的鉴定能力^［9］^。

网络药理学是一门综合性学科^［10］^，其能系统研究药物与生物体的相互作用，突破传统单一靶点的局限，更符合复杂疾病的治疗需求，可分析“成分-靶点-疾病”三者之间的关系，从而探究药物与疾病之间的关联性，为中药的多成分、多靶点作用机制提供科学依据，助力中药现代化研究。分子对接是一种基于计算机模拟的药物设计方法，通过预测小分子配体与大分子蛋白质受体之间的结合模式和亲和力，筛选潜在活性成分并阐释其作用机制。该方法因具有高效性、低成本性和高度指导性等优势，已成为现代药物研发不可或缺的工具。熊竞争等^［11］^利用网络药理学及分子对接技术研究发现，百解胶囊活性成分巴马汀、小檗碱可能作用于转录激活因子3（signal transducer and activator of transcription 3， STAT3）、基质金属蛋白酶9（matrix metalloproteinase-9， MMP9）、哺乳动物雷帕霉素靶蛋白（mammalian target of rapamycin， MTOR）以及细胞间黏附分子1（intercellular adhesion molecule 1， ICAM1）等34个核心蛋白质靶点，通过调控磷脂酰肌醇3-激酶/蛋白激酶B（PI3K/Akt）信号通路、脂质与动脉粥样硬化通路、趋化因子信号通路以及Janus激酶/信号转导及转录激活因子（JAK/STAT）信号通路、钙离子信号通路、瞬时受体电位（transient receptor potential， TRP）通道的炎症介质调节等途径干预湿疹。然而，网络药理学与分子对接所得结果主要来源于数据库预测和计算模拟，仍需通过实验研究进行进一步验证。动物实验能够从整体水平评价药物的药效学作用，并结合炎症因子检测及相关信号通路蛋白表达分析对预测的作用靶点及信号通路进行验证，从而更为全面地阐明药物的药理作用机制^［12］^。因此，将UPLC-LTQ-Orbitrap-MS、网络药理学与动物实验技术相整合，可构建“成分鉴定-靶点预测-机制解析”的系统研究策略，在明确川西合耳菊化学物质基础的同时，从多成分-多靶点-多通路层面揭示其治疗急性湿疹的潜在作用机制。

在此基础上，本研究构建了一种基于UPLC-LTQ-Orbitrap-MS的“成分鉴定-靶点预测-机制解析”的系统研究策略。首先，通过正、负离子模式全扫描获取样品的高分辨质谱信息；其次，结合文献报道、对照品验证以及特征碎片裂解规律，对化合物进行系统鉴定；最后，结合网络药理学、分子对接和动物实验分析验证，探讨川西合耳菊对急性湿疹的治疗作用及其机制。本研究为系统阐明川西合耳菊药效物质基础提供了重要依据，也为其临床应用提供了实验依据。

## 1 实验部分

### 1.1 实验动物

昆明（KM）小鼠，无特定病原体（SPF）级，雄性，共60只，体重18~22 g，由江西中医药大学动物中心提供，合格证号SCXK（赣）2023-0001，本研究使用的所有实验方案均经江西中医药大学动物伦理委员会批准（批准号：JZLLSC20250599）。

### 1.2 仪器

LTQ-Orbitrap Elite高分辨质谱仪、Ultimate 3000超高效液相色谱仪，包含Xcalibur 2.1化学工作站和TraceFinder 4.1软件、Varioskan LUX酶标仪、NanoDrop 2000型超微量分光光度计（美国Thermo Fisher Scientific公司）；BSA224S型十万分之一电子天平（德国Sartorius公司）；KQ-3200DE型数控超声波清洗仪（昆山市超声仪器有限公司）；TGL-16B型高速离心机（上海安亭科学仪器厂）；Milli-Q Synthesis型超纯水纯化系统（美国Millipore公司）；Leica CG1600型包埋机、Leica RM2245型半自动石蜡组织切片机（徳国Leica公司）；重庆重光XDS-1B型生物显微镜（重庆重光实业有限公司）；GENIUS 16-R型台式高速冷冻离心机（美国Beckman Coulter公司）；Tanon 4600SF型凝胶电泳仪（上海天能科技有限公司）；Peiqing JS-780型凝胶图像分析系统（上海培清科技有限公司）；Scilogex SCI1000-G型梯度PCR基因扩增仪（美国Scilogex公司）；Rocgene Archimed S6型实时荧光定量PCR仪（北京罗科基因技术有限公司）。

### 1.3 药物与试剂

标准品：芦丁（批号：JB241985，纯度≥98%）、异槲皮苷（批号：X29011Y12870，纯度≥98%）、绿原酸（批号：Y20A11K11541，纯度≥98%）、苹果酸（批号：Z2656H3962，纯度≥98%）、隐绿原酸（批号：PI6A10U95423，纯度≥98%）、对香豆酸（批号：SN1111GA14，纯度≥98%）均购自上海源叶生物科技有限公司；山柰酚（批号：FY6TBD803，纯度≥98%）、槲皮素（批号：FYI8B406，纯度≥98%）均购自南通飞宇生物科技有限公司；异绿原酸A（批号：202346250，纯度≥98%）购自成都普利斯生物科技有限公司；紫云英苷（批号：23011308，纯度≥98%）购自成都格利普生物科技有限公司；木犀草素（批号：PS010346，纯度≥98%）购自成都普思生物科技有限公司；倒千里光碱（批号：R2406185608，纯度≥98%）购自四川恒诚致远生物科技有限公司。

试剂：实验用水为超纯水；甲醇、乙腈为质谱级，购自德国Merck公司；甲酸为质谱级，购自美国Thermo Fisher公司；2，4-二硝基氯苯（DNCB，批号：UDA2G-QM）购自梯希爱化成工业发展有限公司；丙酮、乙醇、二甲苯均购自西陇科学股份有限公司；橄榄油（批号：0217153）购自上海玻尔化学试剂有限公司；氯化钠注射液（批号：E24032826）购自辰欣药业股份有限公司；苏木素-伊红（HE）染色试剂盒（批号：2311007）、中性树脂（批号：419Y021）、放射免疫沉淀法（RIPA）组织细胞快速裂解液（批号：R0020）、20×含吐温-20的三羟甲基氨基甲烷（TBST）缓冲液（批号：T1082）均购自北京索莱宝科技有限公司；肿瘤坏死因子α（TNF-α）（批号：HB049-Mu）、干扰素γ（IFN-γ）（批号：HB1098-Mu）、白细胞介素6（IL-6）（批号：HB1080-Mu）、IL-17（批号：HB1061-Mu）检测试剂盒均购自上海恒远生物科技有限公司；超纯RNA提取试剂盒（Ultrapure RNA Kit）（批号：CW0581M）、去基因组DNA逆转录预混液（HiFiScript gDNA Removal RT MasterMix）（批号：CW2020M）、SYBR实时荧光定量聚合酶链反应（qRT-PCR）预混液（MagicSYBR Mixture）（批号：CW3008H）、TRIzon总RNA提取试剂（批号：CW0580S）均购自康为世纪生物科技有限公司；4%多聚甲醛（批号：BL539A）、切片石蜡（批号：BL952A）、Super Red核酸染料（批号：BS354B）、DNA上样缓冲液（6×）（批号：BL532B）、二喹啉甲酸（BCA）蛋白定量试剂盒（批号：BL521A）均购自北京兰杰柯科技有限公司；MOPS-SDS Running Buffer（批号：F00004）、FuturePAGE^TM^蛋白预制胶（批号：ET12420Gel）购自ACE公司；PerfectStart Green qPCR SuperMix预混液（批号：AQ601）购自北京全式金生物；多色预染蛋白Marker（批号：WJ101）购自上海雅酶生物医药科技有限公司；丽春红染色液（批号：P0022）、考马斯亮蓝快速染色液（批号：P0017）、QuickBlock Western封闭液（批号：P0252）、一抗稀释液（批号：P0256）、二抗稀释液（批号：P0258）、Western快速转膜液（批号：P0575）均购自碧云天生物科技有限公司；Meilunbio飞克特超敏增强化学发光（ECL）发光液（批号：MA0186）购自Meilunbio公司；其余未注明试剂均为分析纯。

样品：川西合耳菊药材采自西藏拉萨市墨竹贡嘎县，经江西中医药大学药学院龚千峰教授鉴定为菊科合耳菊属植物川西合耳菊*S. solidaginea*的干燥地上部分；复方醋酸地塞米松乳膏（批号：240607）购自广东泰恩康制药厂有限公司。

### 1.4 样品制备

将川西合耳菊药材进行粉碎（过40目筛），精密称取药材0.2 g，加入10 mL的50%甲醇水溶液，超声提取40 min；取上清液于离心管内，放入高速离心机中以13 000 r/min离心10 min；离心后取上清液过0.22 μm微孔滤膜，置于1.5 mL液相瓶内，即得川西合耳菊提取液供试品溶液。

取各对照品适量于10 mL容量瓶中，加50%甲醇溶液定容至刻度，取上清液过0.22 μm微孔滤膜，置于1.5 mL液相瓶内，即得对照品溶液。将川西合耳菊加20倍量水浸泡30 min，煎煮两次，合并滤液，浓缩成高、中、低剂量分别为1.2、1.0、0.8 g/mL，即得小鼠湿疹模型的外用涂抹给药不同剂量组。

### 1.5 仪器条件

#### 1.5.1 色谱条件

ACQUITY UPLC BEH C_18_ 色谱柱（100 mm×2.1 mm，1.7 μm）；流动相：0.1%甲酸水溶液（A）-乙腈（B）；正离子模式下的梯度洗脱程序：0~3 min，5%B； 3~40 min，5%B~68%B； 40~41 min， 68%B~74%B； 41~45 min， 74%B~90%B； 45~47 min， 90%B； 47~47.1 min， 90%B~5%B； 47.1~50 min， 5%B。负离子模式下的梯度洗脱程序：0~5 min，5%B；5~10 min， 5%B~12%B；10~24 min，12%B~22%B；24~28 min，22%B~25%B；28~34 min，25%B~32%B；34~38 min，32%B~34%B；38~39 min，34%B~47%B；39~49 min，47%B~80%B；49~50 min，80%B；50~50.1 min，80%B~5%B； 50.1~52 min，5%B。流速0.3 mL/min，柱温40 ℃，进样量2 μL。

#### 1.5.2 质谱条件

采用电喷雾离子源（ESI），正、负离子模式下离子源温度分别为350 ℃和300 ℃，毛细管温度320 ℃，鞘气流速35 L/h，辅助气流速10 L/h，喷雾电压分别为4 kV和3.6 kV，毛细管电压35 V，管透镜电压110 V，样品先进行全扫描，分辨率设为30 000，扫描范围*m/z* 100~1 500，二级质谱采用动态数据依赖性扫描（data dependent scan， DDS），选取上一级丰度前六强的峰进行碰撞诱导解离（CID）碎片扫描，以离子阱打拿极（dynode）检测。

### 1.6 动物实验方法

#### 1.6.1 分组、造模及给药方法

将小鼠适应性喂养1周后，随机分为空白对照组、模型对照组、阳性药物对照组以及川西合耳菊水煎液高、中、低剂量组，共6组，每组10只。造模前一天对小鼠腹部2 cm×2 cm区域脱毛处理。实验第1、2天除空白对照组外，脱毛部位均采用100 μL以丙酮-橄榄油（4∶1，体积比）配制的质量浓度为50 g/L的DNCB溶液进行致敏；实验第6天对小鼠背部3 cm×3 cm区域进行脱毛处理；实验第7天除空白对照组外，脱毛部位均采用100 μL以丙酮-橄榄油（4∶1，体积比）配制的质量浓度为10 g/L的DNCB溶液进行激发，每隔3天激发1次，共激发6次。空白组给予生理盐水对照。末次造模24 h后观察到小鼠背部皮肤出现红肿、丘疹、渗出、结痂等典型急性湿疹表现，提示造模成功。末次激发后24 h开始给药，空白对照组、模型对照组给予蒸馏水，川西合耳菊水煎液治疗组给予1.2、1.0、0.8 g/mL药液（1 mL/cm^2^），阳性药物对照组给予复方醋酸地塞米松乳膏（1 mg/cm^2^），给药剂量以醋酸地塞米松计为质量分数0.1%，每天2次，连续14 d。

#### 1.6.2 各组小鼠背部急性湿疹皮损程度评分和搔抓次数测定

皮损程度评分采用“湿疹面积及严重度指数”（EASI）评分法。根据红斑、水肿/丘疹、渗出/结痂、抓痕/表皮剥脱4个临床表现的严重程度计分（0~3分），分值越大，症状越严重，分值可记半分（0.5分）。每组小鼠评分取平均值。搔抓次数测定，在末次给药后，将小鼠放置在透明观察笼中，实时记录1 h内小鼠的抓挠次数，统计各组在最后一次造模或最后一次给药后的抓挠次数。

#### 1.6.3 各组小鼠皮损组织标本采集

于末次给药后24 h，采用摘眼球法采集小鼠血液，随后处死小鼠。血液样本静置后离心分离血清，用于酶联免疫吸附测定（enzyme-linked immunosorbent assay， ELISA）检测。处死后，迅速剪取部分背部皮损组织，置于-80 ℃冰箱中冻存，按每50 mg组织加入1 mL TRIzon试剂的比例，于冰浴中充分匀浆裂解，随后按TRIzon试剂说明书进行总RNA提取，所得总RNA经超微量分光光度计测定浓度与纯度后，用于后续逆转录及qRT-PCR检测；另取部分背部皮肤组织，置于4%多聚甲醛溶液中固定，用于HE染色及石蜡切片制备。同时，摘取小鼠脾脏、肝脏及胸腺，称定质量并记录。

#### 1.6.4 各组小鼠脾脏、肝脏和胸腺指数的测定

根据1.6.3节记录的小鼠体质量及各脏器质量，按以下公式分别计算脾脏指数、肝脏指数及胸腺指数：脾脏/肝脏/胸腺指数=脾脏/肝脏/胸腺质量（mg）×1 000/体质量（g）×10。式中，脏器质量与体质量均以1.6.3节称定值为准。

#### 1.6.5 各组小鼠皮肤组织的HE染色病理学检测

将皮肤组织置于体积分数为4%的多聚甲醛溶液中固定24 h，随后依次通过体积分数为70%、80%、95%的乙醇梯度脱水、二甲苯透明化处理后，在60~65 ℃石蜡中浸蜡。组织经石蜡包埋后，切成厚度为5 μm的切片，烤片后进行HE染色。染色步骤如下：组织切片经二甲苯脱蜡，梯度乙醇水化后，进行HE染色。具体染色步骤为苏木精染液染色6 min，盐酸乙醇分化后以自来水返蓝，随后伊红复染3 min，染色后切片经梯度乙醇脱水、二甲苯透明化处理，最后以中性树胶封固。

#### 1.6.6 ELISA方法检测各组小鼠血清中TNF-α、IFN-γ、IL-6、IL-17含量

首先，通过眼球取血获得全血，静置1 h后于4 ℃、3 000 r/min条件下离心15 min，收集上层血清。随后严格按照试剂盒说明进行操作：将标准品按梯度稀释后，与样本分别加入相应微孔中，37 ℃温育30 min。弃去孔内液体，以洗涤液重复洗涤微孔板5次后，加入酶标试剂再次温育，重复洗涤步骤后依次加入显色液A和B，37 ℃避光反应20 min，最后加入终止液。在终止反应15 min内于450 nm波长下测定各孔吸光度值，以标准品浓度为横坐标、吸光度值为纵坐标绘制标准曲线，计算各样本中TNF-α、IFN-γ、IL-6、IL-17的血清浓度。

#### 1.6.7 qRT-PCR检测皮肤组织PI3K、Akt、TNF-α、IL-6、mRNA表达

首先，取-80 ℃冻存皮肤组织，使用TRIzon试剂进行匀浆并提取总RNA。通过1.2%琼脂糖凝胶电泳及超微量分光光度计检测RNA质量，仅OD260/OD280值为1.8~2.2的样本用于后续实验。随后按表1所示逆转录体系将RNA合成cDNA。

**表1:** 逆转录体系

Component	Volume/μL
Total RNA Template 1	3
5×HiFiScript RTMaster Mix	4
10×gDNA Remover Mix	1
RNase-free Water	12
Total	20

实验中所有引物均由上海生工生物工程股份有限公司合成，其碱基序列见表2。

**表2:** 检测所用引物及碱基序列

Primer name	Sequence （5′→3′）	
	Forward	Reverse
PI3K	CGTGCTTTTCAGATTTCCAGCCG	TCCCCAGTACCATTCAGCATCCT
Akt	AAGGACGGTGCCACTATGAA	TCCTGGTTGTAGAAGGGCAG
TNF-α	GGTGCCTATGTCTCAGCCTCTTC	TGATCTGAGTGTGAGGGTCTGGG
IL-6	GGATACCACTCCCAACAGACCTG	TGTTCTTCATGTACTCCAGGTAGCT
GAPDH	GCCCAGAACATCATCCCTGCAT	GCCTGCTTCACCACCTTCTTGA

PI3K： phosphatidylinositol 3-kinase； Akt： protein kinase B （PKB）； TNF-α： tumor necrosis factor-alpha； IL-6： interleukin-6； GAPDH： glyceraldehyde-3-phosphate dehydrogenase.

随后按表3配制PCR反应体系进行扩增。反应程序设置如下：95 ℃预变性2 min；继以95 ℃ 2 s、60 ℃ 34 s，共40个循环；最后采集溶解曲线（65 ℃至95 ℃，每升高0.2 ℃检测一次）。基因相对表达量采用2-ΔΔCT法^［13］^进行计算。该方法基于反应过程中荧光信号达到设定阈值所需的循环数，即CT值（cycle threshold），其数值与初始模板拷贝数成反比。计算时，首先以GAPDH（或其他内参基因）为内参，根据公式ΔCT=CT（目的基因）-CT（内参基因），计算每个样本的ΔCT值；随后，根据公式ΔΔCT=ΔCT（处理组）-ΔCT（对照组），计算各组样本的ΔΔCT值。最后，目的基因的相对表达量以2-ΔΔCT计算，用以表示处理组相较于对照组的表达倍数变化。

**表3:** PCR反应体系

Component	Volume/μL
cDNA	1
Forward Primer （10 μmol/L）	0.4
Reverse Primer （10 μmol/L）	0.4
2×PerfectStart Green qPCR SuperMix	10
Nuclease-free water	8.2
Total	20

#### 1.6.8 Western blot检测皮肤组织中PI3K、p-PI3K、Akt、p-Akt蛋白水平

在含蛋白酶抑制剂的RIPA组织裂解液中将皮肤组织样本充分匀浆裂解，于4 ℃、12 000 r/min条件下离心15 min，收集上清，并使用BCA法测定总蛋白浓度，样品保存于-80 ℃备用。按每孔10 μg蛋白量上样，经沸水浴变性后，使用FuturePAGE蛋白预制胶于160 V恒压下电泳40 min。随后通过湿转法将蛋白转至聚偏二氟乙烯（PVDF）膜上，转膜后以丽春红染色验证转膜效果，用TBST洗净。将膜置于快速封闭液中室温封闭15 min，随后与一抗（目的蛋白一抗稀释比为1∶500，内参抗体稀释比为1∶2 000）在4 ℃下孵育过夜。TBST洗膜3次后，与二抗于37 ℃孵育1 h，再次洗涤。最后使用ECL发光液进行显影，在暗室中曝光并采集图像，通过ImageJ软件分析目标条带的灰度值。

#### 1.6.9 统计学分析

使用SPSS 27.0软件进行数据分析，计量资料采用均值±标准差（mean±SD）表示，进行单因素方差分析，*P<*0.05具有统计学意义。

## 2 结果与讨论

### 2.1 川西合耳菊成分分析

#### 2.1.1 川西合耳菊化学成分分析

通过文献检索收集川西合耳菊的化学成分信息^［14-67］^，建立包含化学成分的中英文名称、分子式、结构类型的川西合耳菊化学成分数据库。采用UPLC-LTQ-Orbitrap-MS对川西合耳菊提取液进行化学成分分析，得到正、负离子模式下的总离子流色谱图，见图1。

**图1:**
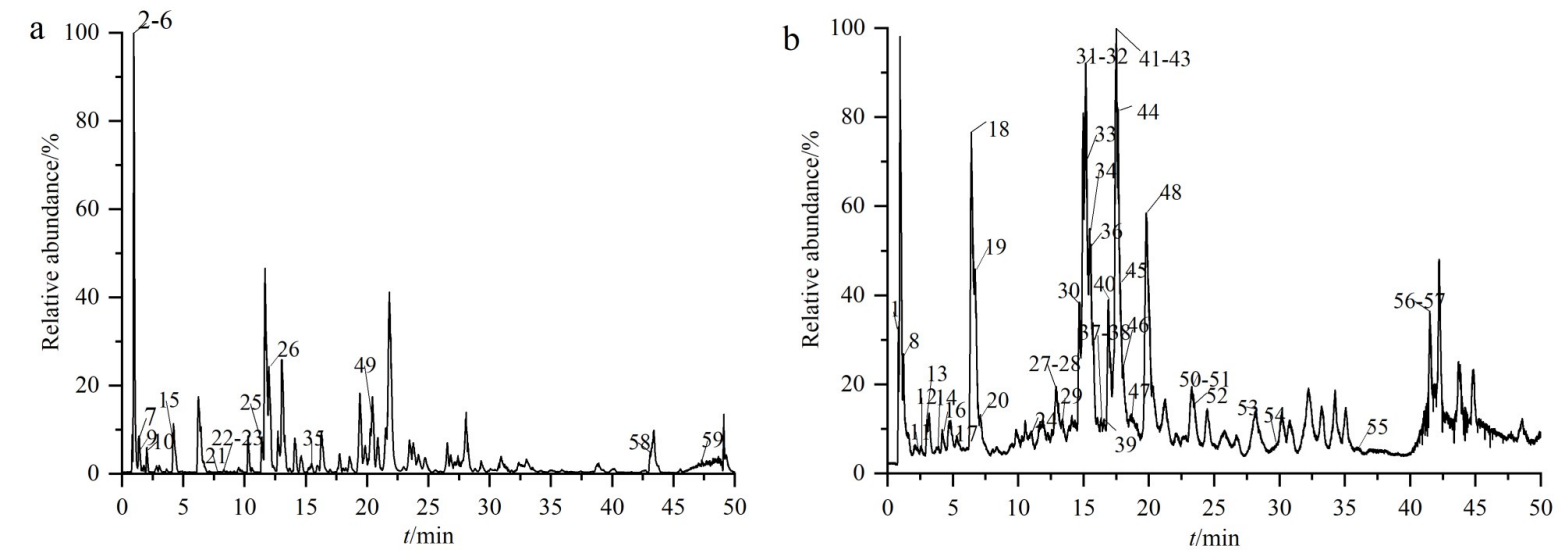
川西合耳菊提取液在（a）正、（b）负离子模式下的总离子流色谱图

采用TraceFinder 4.1软件处理，分析化学成分的保留时间、一级和二级碎片离子信息，初步筛选出质量偏差<5×10^-6^（<5 ppm）的化合物，获取化合物的特征碎片离子，并获取符合条件的准分子离子的二级碎片信息，结合对照品裂解规律及与文献［^15^-^67^］进一步比对，共鉴定出59种化学成分，其中黄酮类20种、酚酸类18种、生物碱类11种，其他类10种，见表4。

**表4:** 川西合耳菊提取液中59种化学成分的分析与鉴定

No.	*t*_R_/min	Formula	Adduct ion	Calculated *m/z*	Observed *m/z*	Mass error/10^-6^	Characteristic fragment ion （*m/z*）	Fragment assignment	Compound	Classification	Refs.
1	0.99	C_4_H_6_O_5_	［M-H］^-^	133.01425	133.01448	1.73	115	［M-H-H_2_O］^-^	malic acid^*^	organic acid	［^15^］
							71	［M-H-CO_2_-H_2_O］^-^			
2	1	C_5_H_5_N_5_	［M+H］^+^	136.06177	136.06113	-4.73	118	［M+H-NH_3_］^+^	adenine	nucleotide	［^16^］
							92	［M+H-NH_3_-HCN］^+^			
3	1.01	C_8_H_13_NO_2_	［M+H］^+^	156.10191	156.10118	-4.67	138	［M+H-H_2_O］^+^	retronecine	alkaloid	［^17^，^18^］
							120	［M+H-H_2_O］^+^			
4	1.02	C_6_H_11_NO_2_	［M+H］^+^	130.08626	130.08571	-4.24	84	［M+H-H_2_O-CO］^+^	pipecolic acid	organic acid	［^19^］
5	1.07	C_5_H_11_NO_2_	［M+H］^+^	118.08626	118.08568	-4.93	59	［M+H-C_3_H_9_N］^+^	betaine	alkaloid	［^20^］
							58	［M+H-C_2_H_2_O_2_］^+^			
6	1.16	C_7_H_13_NO_2_	［M+H］^+^	144.10191	144.10150	-2.84	84	［M+H-C_3_H_10_N］^+^	stachydrine	alkaloid	［^21^，^22^］
							58	［M+H-C_4_H_7_O_2_］^+^			
7	1.25	C_9_H_8_O_3_	［M+H］^+^	165.05462	165.05418	-2.64	121	［M+H-CO_2_］^+^	*p*-coumaric acid^*^	phenolic acid	［^23^］
8	1.48	C_7_H_6_O_5_	［M-H］^-^	169.01425	169.01465	2.36	125	［M-H-CO_2_］^-^	gallic acid	phenolic acid	［^24^］
9	1.76	C_5_H_6_N_2_O_2_	［M+H］^+^	127.05020	127.04977	-3.41	110	［M+H-NH_3_］^+^	thymine	nucleotide	［^16^］
							84	［M+H-HNCO］^+^			
10	2.03	C_9_H_11_NO_2_	［M+H］^+^	166.08626	166.08546	-4.79	120	［M+H-HCOOH］^+^	phenylalanine	amino acid	［^23^］
							103	［M+H-HCOOH-NH_3_］^+^			
11	2.23	C_8_H_8_O_4_	［M-H］^-^	167.03498	167.03537	2.33	152	［M-H-CH_3_］^-^	vanillic acid	phenolic acid	［^25^］
							123	［M-H-CO_2_］^-^			
							108	［M-H-CO_2_-CH_3_］^-^			
12	2.55	C_7_H_6_O_4_	［M-H］^-^	153.01933	153.01961	1.81	109	［M-H-CO_2_］^-^	protocatechuic acid	phenolic acid	［^26^］
							108	［M-H-COOH］^-^			
13	3.01	C_16_H_18_O_9_	［M-H］^-^	353.08781	353.08787	0.12	191	［M-H-C_9_H_6_O_3_］^-^	neochlorogenic acid	phenolic acid	［^27^，^28^］
							179	［M-H-C_7_H_10_O_5_］^-^			
							173	［M-H-C_9_H_6_O_3_-H_2_O］^-^			
							135	［M-H-C_7_H_10_O_5_-CO_2_］^-^			
14	3.9	C_7_H_6_O_3_	［M-H］^-^	137.02442	137.02475	2.41	109	［M-H-CO］^–^	protocatechualdehyde	phenolic acid	［^29^］
15	3.91	C_7_H_6_O_3_	［M+H］^+^	139.03897	139.03850	-3.40	109	［M-H-CO］^–^	4-hydroxybenzoic acid	organic acid	［^30^］
							93	［M-H-CO_2_］^-^			
16	4.11	C_9_H_10_O_3_	［M-H］^-^	165.05572	165.05611	2.34	119	［M-H-OH-CH_3_O］^-^	methyl 4-hydroxyphenylacetate	lipid	［^31^］
							77	［M-H-OH-CH_3_O-CH_3_O］^-^			
17	5.53	C_18_H_16_O_8_	［M-H］^-^	359.07724	359.07770	1.27	197	［M-H-C_9_H_6_O_3_］^-^	rosmarinic acid	phenolic acid	［^27^］
							179	［M-H-C_9_H_8_O_4_］^-^			
							135	［M-H-C_9_H_6_O_3_-CO_2_-H_2_O］^-^			
18	6.42	C_16_H_18_O_9_	［M-H］^-^	353.08781	353.08677	-0.12	191	［M-H-C_9_H_6_O_3_］^-^	chlorogenic acid^*^	phenolic acid	［^32^，^33^］
							179	［M-H-C_7_H_10_O_5_］^-^			
							173	［M-H-C_9_H_6_O_3_-H_2_O］^-^			
							135	［M-H-C_7_H_10_O_5_-CO_2_］^-^			
19	6.77	C_9_H_8_O_4_	［M-H］^-^	179.03498	179.03543	2.52	135	［M-H-CO_2_］^-^	caffeic acid	phenolic acid	［^34^］
							117	［M-H-CO_2_-H_2_O］^-^			
20	7.14	C_16_H_18_O_9_	［M-H］^-^	353.08781	353.08767	-0.14	191	［M-H-C_9_H_6_O_3_］^-^	cryptochlorogenic acid^*^	phenolic acid	［^27^，^35^］
							179	［M-H-C_7_H_10_O_5_］^-^			
							173	［M-H-C_9_H_6_O_3_-H_2_O］^-^			
							135	［M-H-C_7_H_10_O_5_-CO_2_］^-^			
21	7.77	C_18_H_25_NO_6_	［M+H］^+^	352.17546	352.17423	-3.77	138	［M+H-C_10_H_14_O_5_］^+^	senecionine *N*-oxide	alkaloid	［^36^］
							120	［M+H-C_10_H_14_O_5_-H_2_O］^+^			
22	8.02	C_18_H_25_NO_6_	［M+H］^+^	352.17546	352.17413	3.77	138	［M+H-C_10_H_14_O_5_］^+^	retrorsine^*^	alkaloid	［^37^］
							120	［M+H-C_10_H_14_O_5_-H_2_O］^+^			
23	8.28	C_18_H_25_NO_7_	［M+H］^+^	368.17038	368.16885	-4.15	138	［M+H-C_10_H_14_O_6_］^+^	isatidine	alkaloid	［^37^］
							120	［M+H-C_10_H_14_O_6_-H_2_O］^+^			
24	10.26	C_15_H_10_O_8_	［M-H］^-^	317.03029	317.03058	0.91	299	［M-H-H_2_O］^-^	myricetin	flavonoid	［^38^，^39^］
							273	［M-H-CO_2_］^-^			
25	11.48	C_19_H_27_NO_6_	［M+H］^+^	366.19111	366.19906	2.86	168	［M+H-C_10_H_14_O_4_］^+^	senkirkine	alkaloid	［^40^］
							150	［M+H-C_10_H_14_O_4_-H_2_O］^+^			
							122	［M+H-C_10_H_14_O_4_-H_2_O-CO］^+^			
26	12.05	C_19_H_27_NO_6_	［M+H］^+^	366.19111	366.19006	-2.86	168	［M+H-C_10_H_14_O_4_］^+^	neosenkirkine	alkaloid	［^41^］
							150	［M+H-C_10_H_14_O_4_-H_2_O］^+^			
							122	［M+H-C_10_H_14_O_4_-H_2_O-CO］^+^			
27	12.86	C_11_H_12_O_5_	［M-H］^-^	223.06120	223.06140	0.90	208	［M-H-CH_3_］^-^	sinapic acid	phenolic acid	［^41^］
							179	［M-H-CO_2_］^-^			
							163	［M-H-CH_3_-CO_2_］^-^			
							149	［M-H-CH_3_-CO_2_-CH_3_］^-^			
28	12.95	C_27_H_30_O_17_	［M-H］^-^	625.14102	625.14093	-0.14	463	［M-H-C_6_H_10_O_5_］^-^	quercetin-3-*O*-sophoroside	flavonoid	［^42^］
							445	［M-H-C_6_H_10_O_5_-H_2_O］^-^			
							300	［M-H-C_6_H_10_O_5_-C_6_H_10_O_5_-H_2_O］^-^			
29	13.29	C_15_H_12_O_7_	［M-H］^-^	303.05103	303.05130	0.89	285	［M-H-H_2_O］^-^	taxifolin	flavonoid	［^32^］
							241	［M-H-H_2_O-CO_2_］^-^			
30	14.65	C_27_H_30_O_16_	［M-H］^-^	609.14611	609.14624	0.21	301	［M-H-C_6_H_11_O_4_-C_6_H_9_O_5_］^-^	quercetin 3-*O*-robinobioside	flavonoid	［^43^］
							300	［M-H-C_6_H_11_O_4_-C_6_H_9_O_5_-H］^-^			
							151	［M-H-C_6_H_11_O_4_-C_6_H_9_O_5_-C_8_H_5_O_3_］^-^			
							271	［M-H-C_6_H_11_O_4_-C_6_H_9_O_5_-CH_2_O］^-^			
31	14.8	C_21_H_22_O_11_	［M-H］^-^	449.10893	449.10938	0.99	303	［M-H-C_6_H_10_O_4_］^-^	astilbin	flavonoid	［^44^］
							285	［M-H-C_6_H_10_O_4_-H_2_O］^-^			
							179	［M-H-C_6_H_10_O_4_-C_6_H_4_O_3_］^-^			
32	14.98	C_21_H_20_O_12_	［M-H］^-^	463.08820	463.08700	-0.87	301	［M-H-C_6_H_10_O_5_］^-^	hyperoside	flavonoid	［^45^］
							300	［M-H-C_6_H_10_O_5_-H］^-^			
							271	［M-H-C_6_H_10_O_5_-H-CO］^-^			
33	15.21	C_27_H_30_O_16_	［M-H］^-^	609.14611	609.14625	-0.21	301	［M-H-C_12_H_20_O_9_］^-^	rutin^*^	flavonoid	［^46^］
							300	［M-H-C_12_H_20_O_9_-H］^-^			
							271	［M-H-C_12_H_20_O_9_-CH_2_O］^-^			
							151	［M-H-C_12_H_20_O_9_-C_8_H_5_O_3_］^-^			
34	15.55	C_21_H_20_O_12_	［M-H］^-^	463.08820	463.08780	-0.87	301	［M-H-C_6_H_10_O_5_］^-^	quercetin-7-*O*-*β*-D-glucopyranoside	flavonoid	［^47^］
							300	［M-H-C_6_H_10_O_5_-H］^-^			
							271	［M-H-C_6_H_10_O_5_-H-CO］^-^			
35	15.66	C_15_H_12_O_6_	［M+H］^+^	289.07066	289.06979	-3.00	271	［M+H-H_2_O］^+^	aromadendrin	flavonoid	［^48^］
							243	［M+H-H_2_O-CO］^+^			
36	15.71	C_21_H_20_O_12_	［M-H］^-^	463.08820	463.08880	0.87	301	［M-H-C_6_H_10_O_5_］^-^	isoquercitrin^*^	flavonoid	［^49^，^50^］
							300	［M-H-C_6_H_10_O_5_-H］^-^			
							271	［M-H-C_6_H_10_O_5_-H-CO］^-^			
37	16.05	C_17_H_20_O_9_	［M-H］^-^	367.10346	367.10394	1.32	191	［M-H-C_10_H_8_O_3_］^-^	methyl chlorogenate	phenolic acid	［^51^，^52^］
							179	［M-H-C_8_H_12_O_3_］^-^			
							135	［M-H-C_9_H_12_O_7_］^-^			
38	16.35	C_27_H_30_O_15_	［M-H］^-^	593.15119	593.15179	1.02	285	［M-H-C_12_H_20_O_9_］^-^	nicotiflorin	flavonoid	［^53^］
							255	［M-H-C_12_H_20_O_9_-CO-2H］^-^			
							151	［M-H-C_12_H_20_O_9_-C_8_H_6_O_2_］^-^			
39	16.91	C_21_H_20_O_11_	［M-H］^-^	447.09328	447.09252	0.25	285	［M-H-C_9_H_6_O_3_］^-^	kaempferol-3-*O*-galactoside	flavonoid	［^54^］
							284	［M-H-C_9_H_6_O_3_-H］^-^			
							255	［M-H-C_9_H_6_O_3_-H-HCO］^-^			
40	17	C_10_H_10_O_4_	［M-H］^-^	193.05063	193.05099	1.89	178	［M-H-CH_3_］^-^	ferulic acid	phenolic acid	［^25^］
							149	［M-H-CO_2_］^-^			
							134	［M-H-CH_3_-CO_2_］^-^			
41	17.49	C_16_H_18_O_9_	［M-H］^-^	353.08781	353.08777	-0.12	191	［M-H-C_9_H_6_O_3_］^-^	1-caffeoylquinic acid	phenolic acid	［^26^，^55^］
							179	［M-H-C_7_H_10_O_5_］^-^			
							173	［M-H-C_9_H_6_O_3_-H_2_O］^-^			
							135	［M-H-C_7_H_10_O_5_-CO_2_］^-^			
42	17.5	C_25_H_24_O_12_	［M-H］^-^	515.11950	515.11948	-0.22	353	［M-H-C_9_H_6_O_3_］^-^	isochlorogenic acid A^*^	phenolic acid	［^35^，^45^］
							197	［M-H-C_9_H_6_O_3_-C_9_H_6_O_3_］^-^			
							179	［M-H-C_9_H_6_O_3_-C_7_H_10_O_5_］^-^			
							173	［M-H-C_9_H_6_O_3_-C_9_H_6_O_3_-H_2_O］^-^			
43	17.54	C_27_H_30_O_15_	［M-H］^-^	593.15119	593.15109	1.03	285	［M-H-C_12_H_20_O_9_］^-^	lonicerin	flavonoid	［^56^，^57^］
44	17.66	C_8_H_8_O_2_	［M-H］^-^	135.04515	135.04547	2.38	107	［M-H-CO］^-^	phenylacetic acid	organic acid	［^58^］
							91	［M-H-CO_2_］^-^			
45	17.84	C_21_H_20_O_11_	［M-H］^-^	447.09328	447.09317	-0.25	285	［M-H-C_9_H_6_O_3_］^-^	astragalin^*^	flavonoid	［^35^］
							284	［M-H-C_9_H_6_O_3_-H］^-^			
							255	［M-H-C_9_H_6_O_3_-H-HCO］^-^			
							151	［M-H-C_9_H_6_O_3_-C_7_H_2_O_3_］^-^			
46	18.12	C_22_H_22_O_12_	［M-H］^-^	477.10385	477.10370	-0.32	315	［M-H-C_6_H_10_O_5_］^-^	isorhamnetin-3-*O*-*β*-D-glucoside	flavonoid	［^54^］
							300	［M-H-C_6_H_10_O_5_-CH_3_］^-^			
47	18.42	C_11_H_12_O_4_	［M-H］^-^	207.06628	207.06660	1.57	161	［M-H-C_2_H_5_O］^-^	ethyl caffeate	phenolic acid	［^35^］
							135	［M-H-C_3_H_3_O_2_］^-^			
							133	［M-H-C_2_H_5_O-CO］^-^			
48	19.8	C_25_H_24_O_12_	［M-H］^-^	515.11950	515.11938	-0.23	353	［M-H-C_9_H_6_O_3_］^-^	isochlorogenic acid C	phenolic acid	［^35^］
							197	［M-H-C_9_H_6_O_3_-C_9_H_6_O_3_］^-^			
							179	［M-H-C_9_H_6_O_3_-C_7_H_10_O_5_］^-^			
							173	［M-H-C_9_H_6_O_3_-C_9_H_6_O_3_-H_2_O］^-^			
49	20.25	C_15_H_20_O_3_	［M+H］^+^	249.14852	249.14781	-2.84	231	［M-H_2_O］^+^	tomentosin	terpenoids	［^59^］
50	23.23	C_15_H_10_O_7_	［M-H］^-^	301.03538	301.03540	0.07	273	［M-H-CO］^-^	quercetin^*^	flavonoid	［^22^］
							179	［M-H-C_6_H_2_O_3_］^-^			
							151	［M-H-C_8_H_6_O_3_］^-^			
51	23.34	C_21_H_20_O_9_	［M-H］^-^	415.10346	415.10388	1.02	295	［M-H-C_4_H_8_O_4_］^-^	puerarin	flavonoid	［^23^］
							267	［M-H-C_4_H_8_O_4_-CO］^-^			
52	23.71	C_15_H_10_O_6_	［M-H］^-^	285.04046	285.04074	0.99	241	［M-H-CO_2_］^-^	luteolin^*^	flavonoid	［^48^，^60^］
							199	［M-H-C_4_H_6_O_2_］^-^			
							151	［M-H-C_8_H_6_O_2_］^-^			
53	28.23	C_15_H_10_O_6_	［M-H］^-^	285.04046	285.04034	-0.99	257	［M-H-CO］^-^	kaempferol^*^	flavonoid	［^48^，^60^］
							229	［M-H-2CO］^-^			
							185	［M-H-C_4_H_4_O_3_］^-^			
							169	［M-H-C_4_H_4_O_3_］^-^			
							151	［M-H-C_4_H_4_O_3_-H_2_O］^-^			
54	29.54	C_16_H_12_O_7_	［M-H］^-^	315.05103	315.05136	1.05	300	［M-H-CH_3_］^-^	isorhamnetin	flavonoid	［^48^，^61^］
							271	［M-H-CH_3_-CHO］^-^			
							243	［M-H-CH_3_-CHO-CO］^-^			
							151	［M-H-CH_3_-CHO-C_7_H_5_O_2_］^-^			
55	36.87	C_10_H_10_O_4_	［M-H］^-^	193.05063	193.05099	1.89	134	［M-H-CH_3_-CO_2_］^-^	methyl caffeate	phenolic acid	［^62^，^63^］
56	41.84	C_15_H_18_O_3_	［M-H］^-^	245.11832	245.11867	1.42	229	［M-H-O］^-^	xanthatin	terpenoids	［^64^］
							183	［M-H-C_2_H_6_O］^-^			
							159	［M-H-C_2_H_6_O-CO］^-^			
57	41.93	C_27_H_28_N_2_O_4_	［M-H］^-^	443.19763	443.19815	1.17	383	［M-H-C_2_H_4_O_2_］^-^	aurantiamide acetate	alkaloid	［^65^］
58	42.53	C_18_H_35_NO	［M+H］^+^	282.27914	282.27802	-3.98	265	［M+H-NH_3_］^+^	oleamide	alkaloid	［^66^，^67^］
							247	［M+H-NH_3_-H_2_O］^+^			
59	47.03	C_22_H_43_NO	［M+H］^+^	338.34174	338.34033	-4.16	321	［M+H-NH_3_］^+^	erucamide	alkaloid	［^66^，^67^］
							303	［M+H-NH_3_-H_2_O］^+^			

* Confirmed by comparison with the reference standard.

#### 2.1.2 川西合耳菊主要成分的质谱裂解途径分析

##### 2.1.2.1 黄酮类成分

川西合耳菊中黄酮类成分主要为黄酮类、黄酮醇类及其苷类。黄酮苷元类化合物在MS裂解过程中容易丢失CO、CO_2_和CH_3_等中性碎片，或者母核C环发生RDA（retro-Diels-Alder）反应裂解开环，形成A环和B环特征碎片；黄酮苷类化合物则为丢失糖基而形成的丰度较高的黄酮苷元^［15］^。

化合物52与化合物53互为同分异构体，化学式均为C_15_H_10_O_6_，保留时间分别为23.71 min和28.23 min，准分子离子峰分别为*m/z* 285.040 74 ［M-H］^-^和*m/z* 285.040 34 ［M-H］^-^，两者结构相似，均会发生C环RDA裂解生成*m/z* 151碎片离子，而化合物52在负离子模式下分子可失去一分子CO_2_生成*m/z* 241.050 69 ［M-H-CO_2_］^-^碎片离子；分子C环发生RDA裂解失去一分子C_8_H_6_O_2_生成*m/z* 151.003 73 ［M-H-C_8_H_6_O_2_］^-^碎片离子和*m/z* 133.029 54 ［M-H-C_8_H_8_O_2_］^-^碎片离子（C_8_H_5_O_2_^-^）。依据文献［^68^］与对照品可确定，化合物52为木犀草素，化合物53为山柰酚。木犀草素可能的裂解规律见图2。

**图2:**
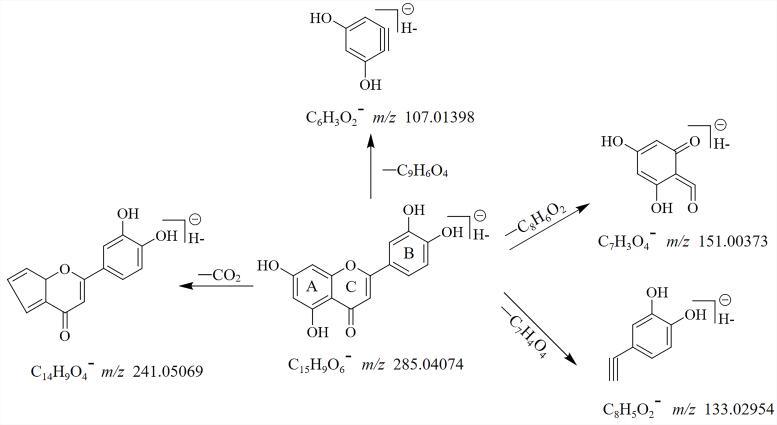
木犀草素可能的裂解规律

化合物39与化合物45为一对同分异构体，化学式均为C_21_H_20_O_11_，保留时间分别为16.91 min和17.84 min，准分子离子峰分别为*m/z* 447.092 52 ［M-H］^-^和*m/z* 447.093 17 ［M-H］^-^。两者分子均易发生糖苷键断裂，失去一分子葡萄糖基（C₆H₁₀O₅）而形成丰度高的黄酮苷元碎片离子，该苷元碎片表现为*m/z* 284.032 61 ［M-H-C_6_H_10_O_5_］^-^（对应C₁₃H₈O₆^-^）与*m/z* 285.040 01 ［M-H-C₆H₁₀O₅］^-^（对应C₁₅H₉O₆^-^），生成的黄酮苷元也会发生裂解生成特征碎片^［35］^。依据文献［^35^］与对照品可确定，化合物39为山柰酚-3-*O*-半乳糖苷，化合物45为紫云英苷。紫云英苷可能的裂解规律见图3。

**图3:**
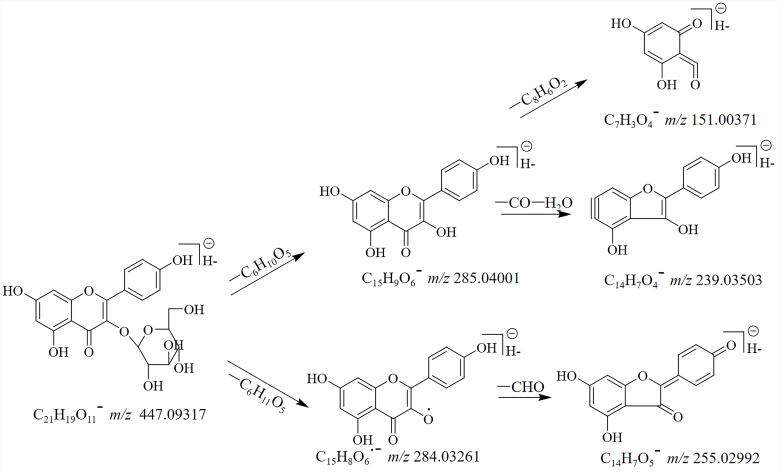
紫云英苷可能的裂解规律

##### 2.1.2.2 酚酸类成分

酚酸类是一类含有酚环的有机酸的化合物，一般在苯环上会有多个酚羟基，结构上含有羧基、甲氧基等基团，通常在MS裂解过程中失去H_2_O、CO、CO_2_等中性碎片，形成特征碎片^［69］^。化合物13、18和20分子式均为C_16_H_18_O_9_，在负离子模式下准分子离子峰分别为*m/z* 353.087 87 ［M-H］^-^、*m/z* 353.086 77 ［M-H］^-^和*m/z* 353.087 67 ［M-H］^-^，保留时间分别为3.01、6.42和7.14 min，均出现*m/z* 191.056 27 ［C_7_H_11_O₆］^-^（对应奎尼酸部分）、*m/z* 179.035 14 ［C₉H₇O₄］^-^（对应咖啡酸部分）、*m/z* 173.045 76 ［C_7_H_9_O_5_］^-^及*m/z* 135.045 36 ［C₈H₇O₂］^-^碎片离子，根据文献［^32^，^33^］和对照品可确定化合物13为新绿原酸，化合物18为绿原酸，化合物20为隐绿原酸。绿原酸可能的裂解规律见图4。

**图4:**
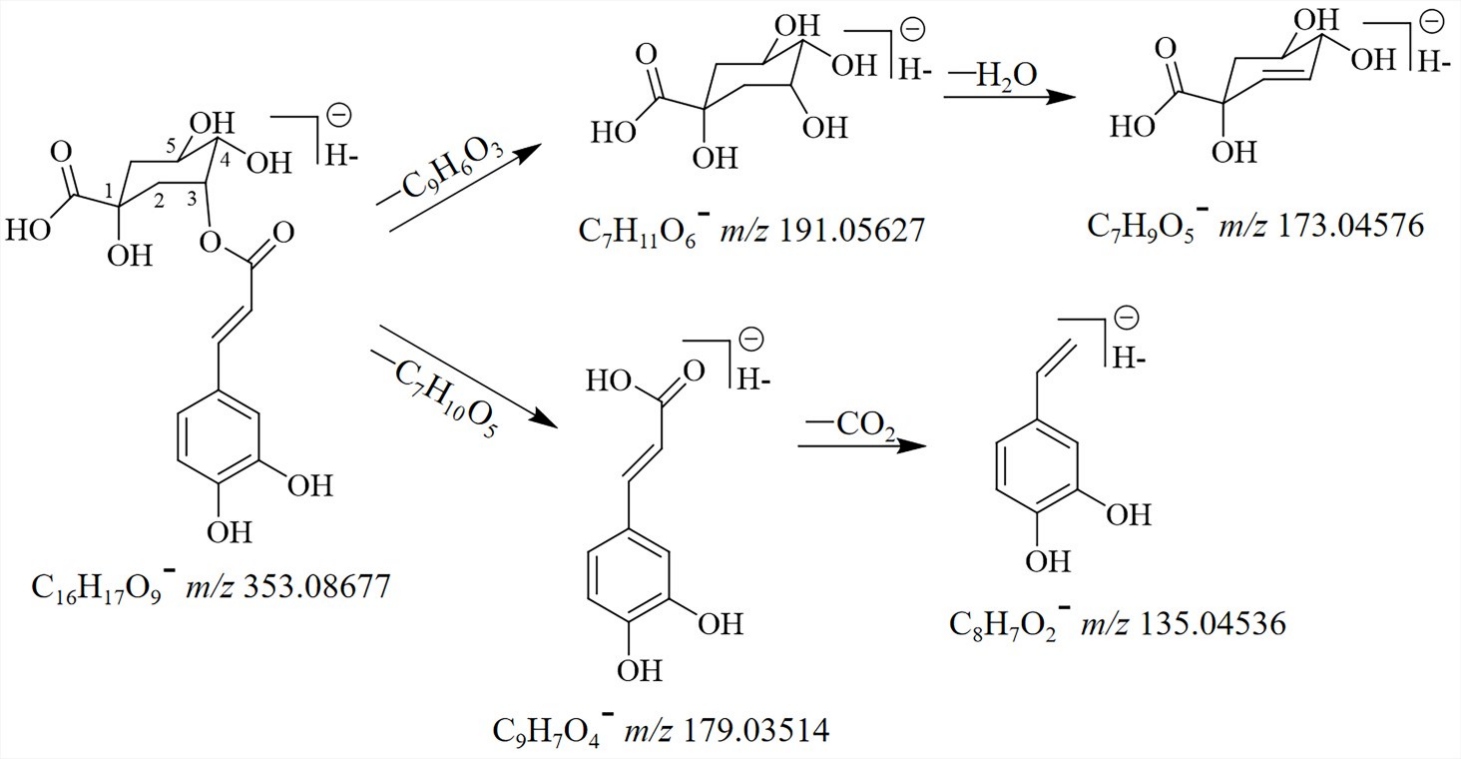
绿原酸可能的裂解规律

化合物42和化合物48为一对同分异构体，分子式均为C_25_H_24_O_12_，保留时间分别为17.5 min和19.8 min，在负离子模式下准分子离子峰分别为*m/z* 515.119 48 ［M-H］^-^和*m/z* 515.119 38 ［M-H］^-^，均出现*m/z* 353.087 91 ［C₁₆H₁₇O₉］^-^（对应丢失一分子咖啡酰基后的绿原酸部分）、*m/z* 191.056 34 ［C₇H₁₁O₆］^-^（奎尼酸部分）、*m/z* 179.035 12 ［C₉H₇O₄］^-^（咖啡酸部分）以及*m/z* 173.045 59 ［C₇H₉O₅］^-^碎片离子，根据文献［^35^］和对照品可确定化合物42为异绿原酸A，化合物48为异绿原酸C）。异绿原酸C可能的裂解规律见图5。

**图5:**
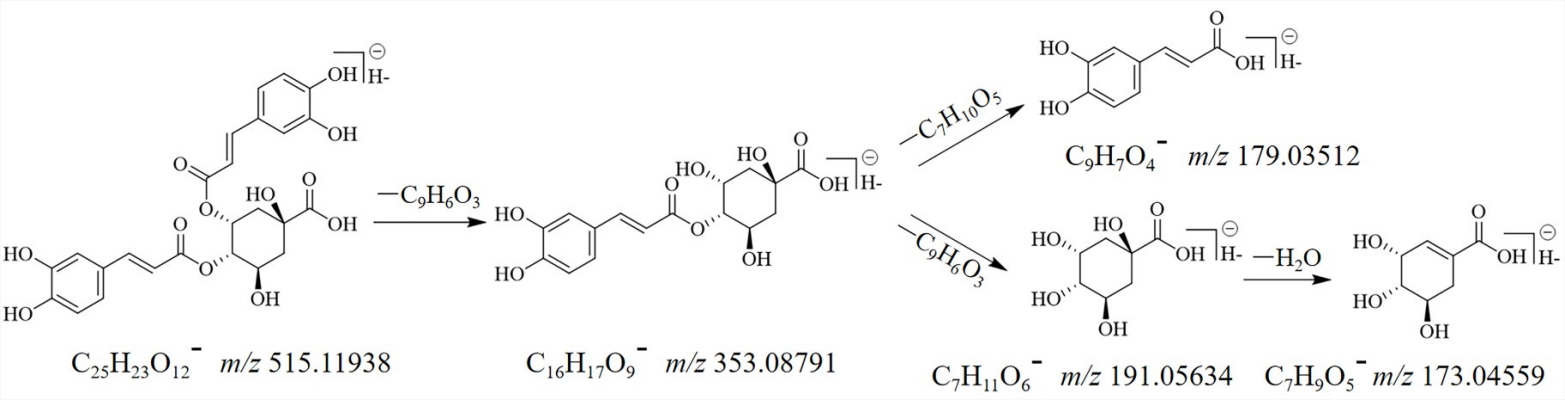
异绿原酸C可能的裂解规律

##### 2.1.2.3 生物碱类成分

生物碱是一类含氮的碱性化合物，在裂解过程中易失去氮及其连接的取代基，吡咯里西啶生物碱是菊科常见的一类生物碱，川西合耳菊也含有此类生物碱。化合物5的分子式为C_5_H_11_NO_2_，保留时间为1.07 min，在正离子模式下准分子离子峰为*m/z* 118.085 68 ［M+H］^+^，分子失去一分子C_3_H_9_N产生*m/z* 59.080 79 ［M+H-C_3_H_9_N］^+^碎片离子，分子失去一分子C_2_H_2_O_2_产生*m/z* 58.091 24 ［M+H-C_2_H_2_O_2_］^+^碎片离子。根据文献［^20^］及其产生的离子峰信息可确定化合物5为甜菜碱（betaine）。

化合物22的化学式为C_18_H_25_NO_6_，保留时间为8.02 min，在正离子模式下准分子离子峰为*m/z* 352.174 13 ［M+H］^+^（对应C₁₈H₂₆NO_6_⁺），分子失去一分子C_10_H_14_O_5_产生*m/z* 138.091 34 ［M+H-C_8_H_12_NO］^+^碎片离子，该碎片进一步失去一分子H_2_O产生*m/z* 120.080 86 ［M+H-C₈H₁₀N］^+^碎片离子，此外，母核还可直接裂解产生*m/z* 94.065 13 ［C₆H₈N］⁺碎片离子。根据文献［^37^］及对照品的信息可确定化合物22为倒千里光碱。化合物3、6、21、25及26，产生的碎片离子与化合物22相似，根据文献［^17^，^18^］及其产生的离子峰信息可确定化合物3为倒千里光碱，化合物6为*N*-氧化千里光宁碱，化合物21为倒千里光碱氮氧化物，化合物25和26为一对克氏千里光碱同分异构体。倒千里光碱可能的裂解规律见图6。

**图6:**
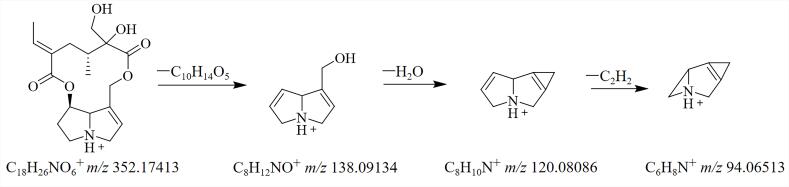
倒千里光碱可能的裂解规律

##### 2.1.2.4 其他类成分

除上述黄酮类、酚酸类、生物碱类外，从川西合耳菊还鉴定出4种有机酸、2种核苷酸、1种氨基酸、2种萜类和1种酯类。

### 2.2 网络药理学及分子对接研究

#### 2.2.1 活性成分筛选

基于UPLC-LTQ-Orbitrap-MS方法分析得到的川西合耳菊化学成分，利用中药系统药理学数据库与分析平台（TCMSP）数据库，以*M*_r_（相对分子质量）<500、DL（生物利用度）≥0.18、1<*A*lg *P*（atomic-based partition coefficient，原子贡献法计算的脂水分配系数）<3为筛选条件^［70］^，筛选川西合耳菊的活性成分，共得到7种活性成分：异鼠李素、山柰酚、木犀草素、槲皮素、杨梅素、甜菜碱和迷迭香酸。将得到的活性成分通过SwissTargetPrediction数据库进行潜在作用蛋白反向预测，删除重复值，得到192个川西合耳菊活性成分的候选靶标蛋白。

#### 2.2.2 急性湿疹疾病靶点

在Gene Cards和在线人类孟德尔遗传（Online Mendelian Inheritance in Man， OMIM）数据库中输入急性湿疹“Acute eczema”检索相关的靶点，获取的全部靶点进行整理，整合归纳删除重复项，得到2 214个急性湿疹疾病关联基因。

#### 2.2.3 交集靶点的蛋白相互作用

采用Venny2.1.0绘制川西合耳菊成分调控靶点与急性湿疹疾病靶点的韦恩图，两者的交集靶点即可能为川西合耳菊治疗急性湿疹的潜在作用靶点（75个），见图7。将上述75个潜在作用靶点导入STRING数据库中，物种限定为“智人”（Homo sapiens），设置置信度评分阈值为0.4，获取靶点间的相互作用关系数据，将STRING数据库导出的相互作用数据文件导入到Cytoscape3.10.1软件中进行可视化，构建蛋白质-蛋白质相互作用（protein-protein interaction，PPI）网络图，对得到的网络图相关数据进一步分析，按度中心性（degree）的大小由高至低排序，筛选出川西合耳菊治疗急性湿疹核心靶点，前10个核心靶点分别为蛋白激酶Bα（Akt serine/threonine kinase 1， Akt1）、表皮生长因子受体（epidermal growth factor receptor， EGFR）、胱天蛋白酶3（caspase-3， CASP3）、原癌基因酪氨酸蛋白激酶SRC（SRC proto-oncogene， non-receptor tyrosine kinase）、MMP9、前列腺素内过氧化物合酶2（prostaglandin-endoperoxide synthase 2，PTGS2）、热休克蛋白90α家族A类成员1（heat shock protein 90 alpha family class A member 1， HSP90AA1）、过氧化物酶体增殖物激活受体γ（peroxisome proliferator-activated receptor gamma，PPARG）、erb-b2受体酪氨酸激酶2（erb-b2 receptor tyrosine kinase 2， ERBB2）和激酶插入结构域受体（kinase insert domain receptor， KDR），见图8。

**图7:**
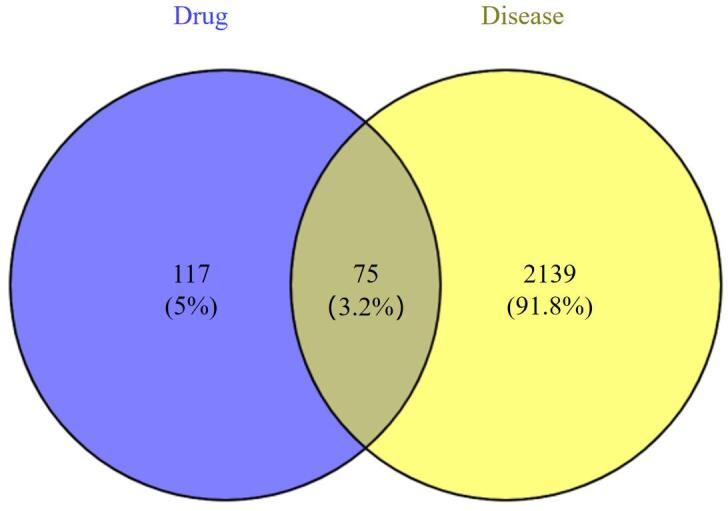
川西合耳菊成分靶点与急性湿疹疾病靶点的韦恩图

**图8:**
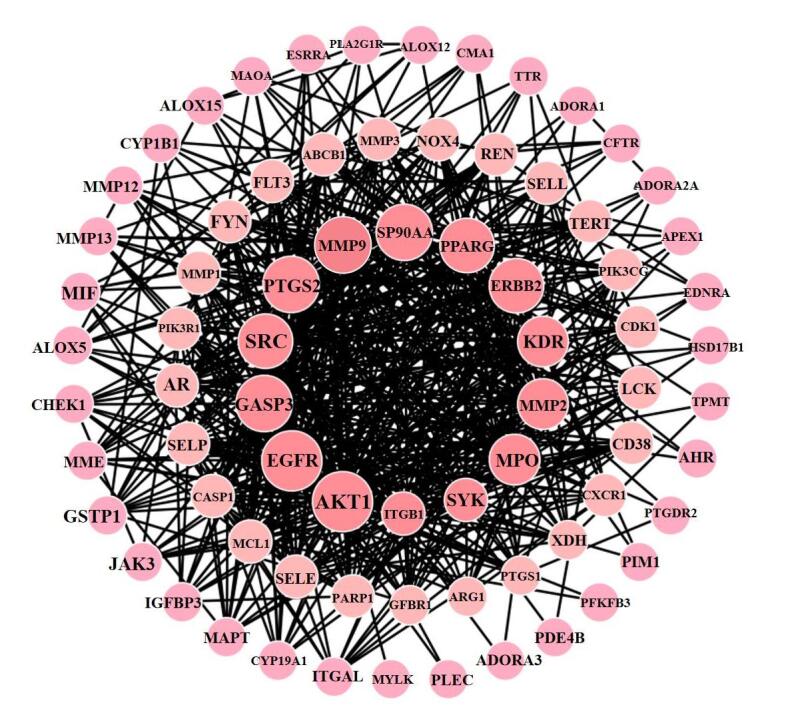
交集靶点的PPI网络图

#### 2.2.4 GO和KEGG富集分析

在DAVID数据库（Database for Annotation， Visualization and Integrated Discovery）中输入PPI网络图中75个潜在作用靶点，进行基因本体论（Gene Ontology，GO）和京都基因与基因组百科全书（Kyoto Encyclopedia of Genes and Genomes，KEGG）分析，再通过微生信在线数据分析平台（上海纽科生物科技有限公司，http：//www.bioinformatics.com.cn）进行可视化。GO分析结果按-lg *P*值进行排名，筛选出排名前10的条目，富集结果可视化见图9。川西合耳菊在治疗急性湿疹的生物过程中与肽酪氨酸磷酸化、凋亡过程的负调控、对外源性刺激的反应等关系密切；在细胞组分分子功能中与细胞膜、核周质膜、细胞质等关系密切；在分子功能中与内肽酶活性、蛋白酪氨酸激酶活性、酶结合相关。

**图9:**
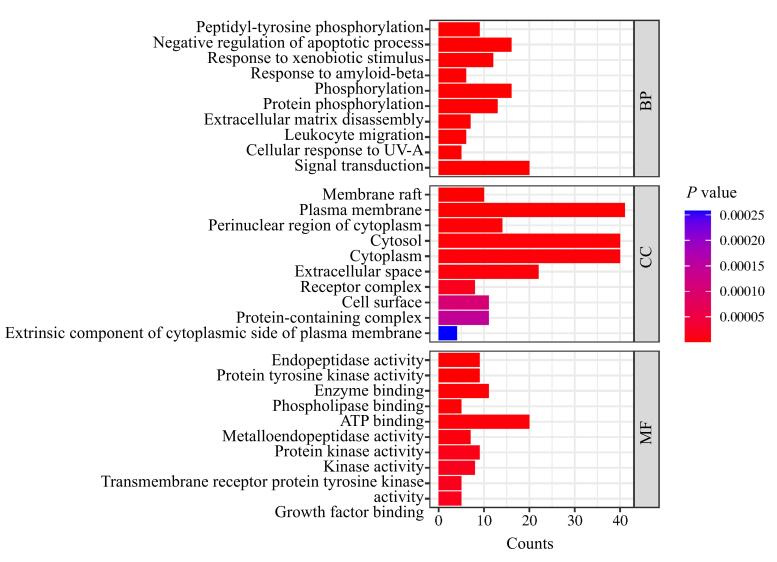
川西合耳菊候选靶标蛋白与急性湿疹疾病关联基因交集的GO功能富集分析

KEGG分析结果按照重叠值、富集值和*P*值筛选，富集分析得到的通路结果根据-lg *P*值和每条通路所连接的基因个数由大到小进行排名，筛选出前20条通路，富集结果可视化见图10，发现富集到前20条通路中，糖尿病并发症中的AGE-RAGE信号通路（AGE-RAGE signaling pathway in diabetic complications）、IL-17信号通路、PI3K-Akt信号通路、TNF信号通路、血管内皮生长因子（vascular endothelial growth factor，VEGF）信号通路、丝裂原活化蛋白激酶（mitogen-activated protein kinase，MAPK）信号通路、JAK-STAT信号通路、核因子kappa B（nuclear factor kappa B，NF-kappa B）信号通路等与急性湿疹关系比较密切^［71］^。从以上结果发现，川西合耳菊抗急性湿疹的主要通路富集在PI3K-Akt等信号通路。PI3K-Akt信号通路作为抗湿疹中重要的信号转导通路，可调控细胞增殖、代谢及凋亡等关键生命活动，还可调控机体组织的氧化还原反应平衡、炎症反应信号的传导^［72］^。在湿疹发生时，Akt还可增强细胞黏附分子的表达，加强了免疫细胞与血管内皮之间的相互作用，并促进炎症细胞向炎症部位的迁移。这些线索进一步表明川西合耳菊可能通过调节PI3K-Akt信号通路抑制炎症、调节免疫细胞而起到抗湿疹的作用。

**图10:**
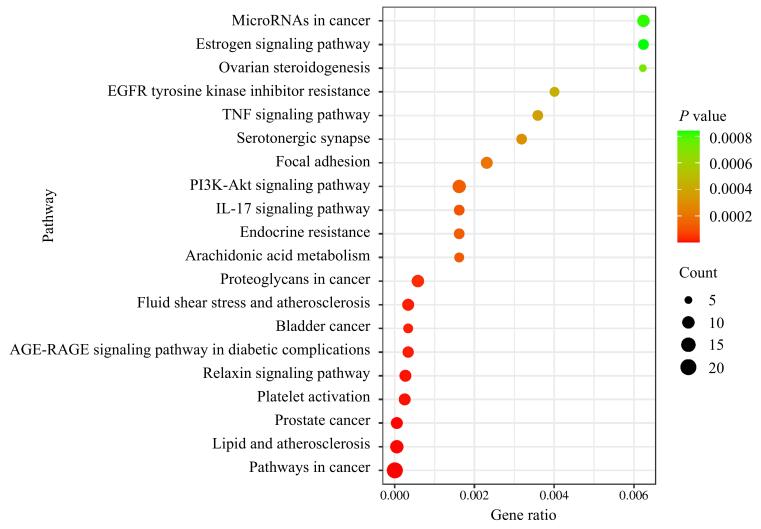
川西合耳菊治疗急性湿疹潜在作用靶点前20条KEGG通路富集气泡图

#### 2.2.5 成分-靶点-通路网络图构建

根据KEGG富集分析的结果，选择排名前20的通路构建“药物成分-靶点-通路”网络图，见图11，药物成分网络节点相关信息表见表5。网络图中三角形节点、椭圆形节点和正方形节点分别代表通路、靶点和药材成分。结合Degree值与介数中心性（betweenness centrality，BC）值的大小和与核心靶点（Akt1、EGFR、SRC、CASP3、MMP9、PTGS2）对应3个及以上的活性成分，筛选出的5种成分均需要关注。

**图11:**
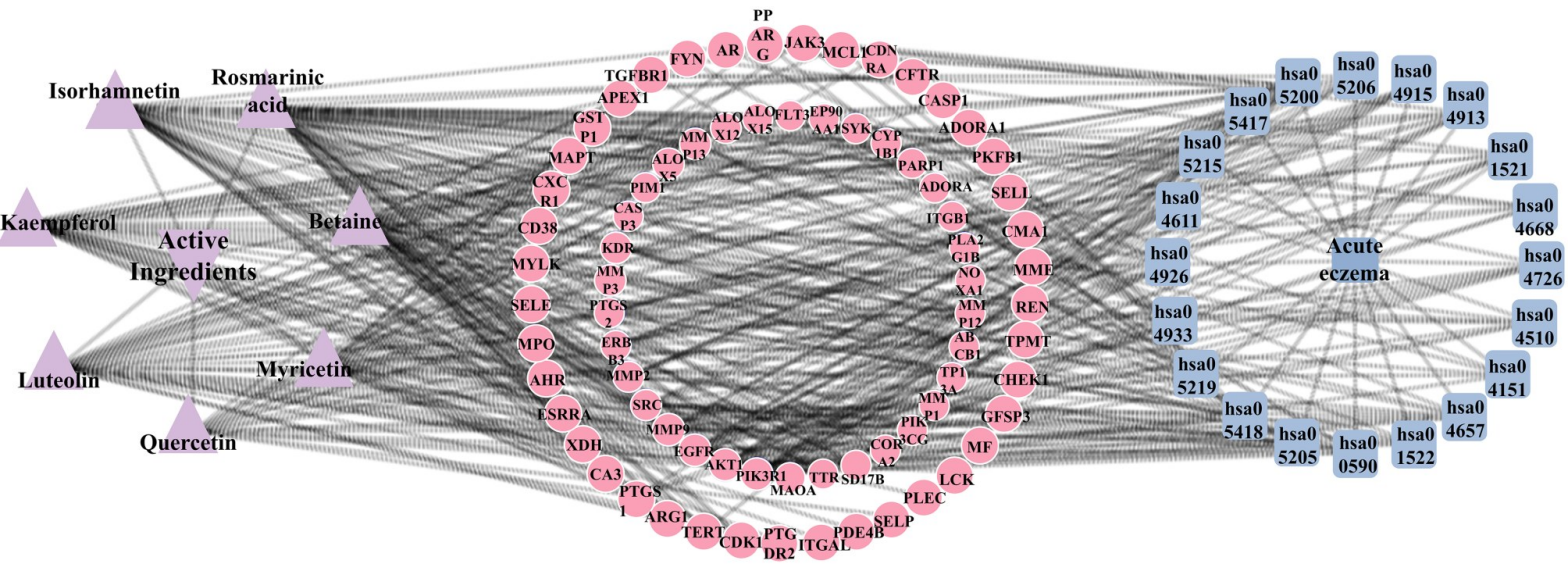
川西合耳菊成分-靶点-通路图

**表5:** 川西合耳菊成分网络节点相关信息

Compound	Core targets	BC	Degree
Isorhamnetin	Akt1， EGFR， SRC， MMP9	0.08	43
Kaempferol	Akt1， EGFR， SRC， MMP9， PTGS2	0.08	42
Luteolin	Akt1， EGFR， SRC， MMP9， PTGS2	0.08	42
Quercetin	Akt1， EGFR， SRC， MMP9	0.07	42
Myricetin	Akt1， EGFR， SRC， MMP9	0.07	42
Betaine	EGFR， PTGS2， CASP3	0.18	30
Rosmarinic acid	EGFR， MMP9	0.10	20

BC： betweenness centrality； Akt1： Akt serine/threonine kinase 1； EGFR： epidermal growth factor receptor； SRC： SRC proto-oncogene， non-receptor tyrosine kinase； MMP9： matrix metalloproteinase-9； PTGS2： prostaglandin-endoperoxide synthase 2； CASP3： caspase-3.

#### 2.2.6 核心靶点与关键成分的分子对接

根据网络药理学分析结果，小分子配体选取Degree值排名前5的药物成分即异鼠李素、山柰酚、木犀草素、槲皮素、杨梅素，大分子受体选取Degree值排名前5的核心靶标蛋白即Akt1、EGFR、SRC、MMP9、PTGS2。从TCMSP数据库中得到小分子配体结构，从PDB蛋白序列数据库得到蛋白质结构，使用AutoDock软件进行分子对接，Pymol软件对分子对接结果进行可视化。川西合耳菊的5个关键成分与核心靶蛋白对接结果见图12，全部关键成分与核心靶蛋白对接的结合能都<-5 kcal/mol（1 kcal=4.184 kJ），说明5个关键成分可以良好地与受体蛋白结合。各成分与核心蛋白结合最强的对接可视化见图13。

**图12:**
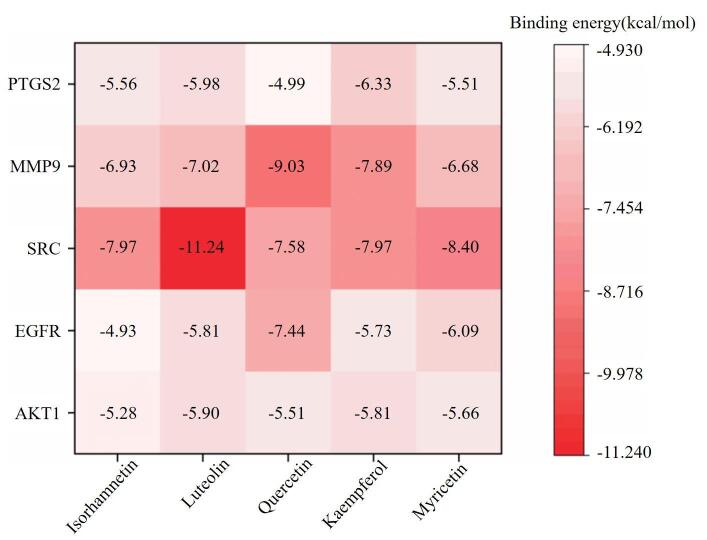
川西合耳菊关键成分与急性湿疹核心靶点蛋白对接结果

**图13:**
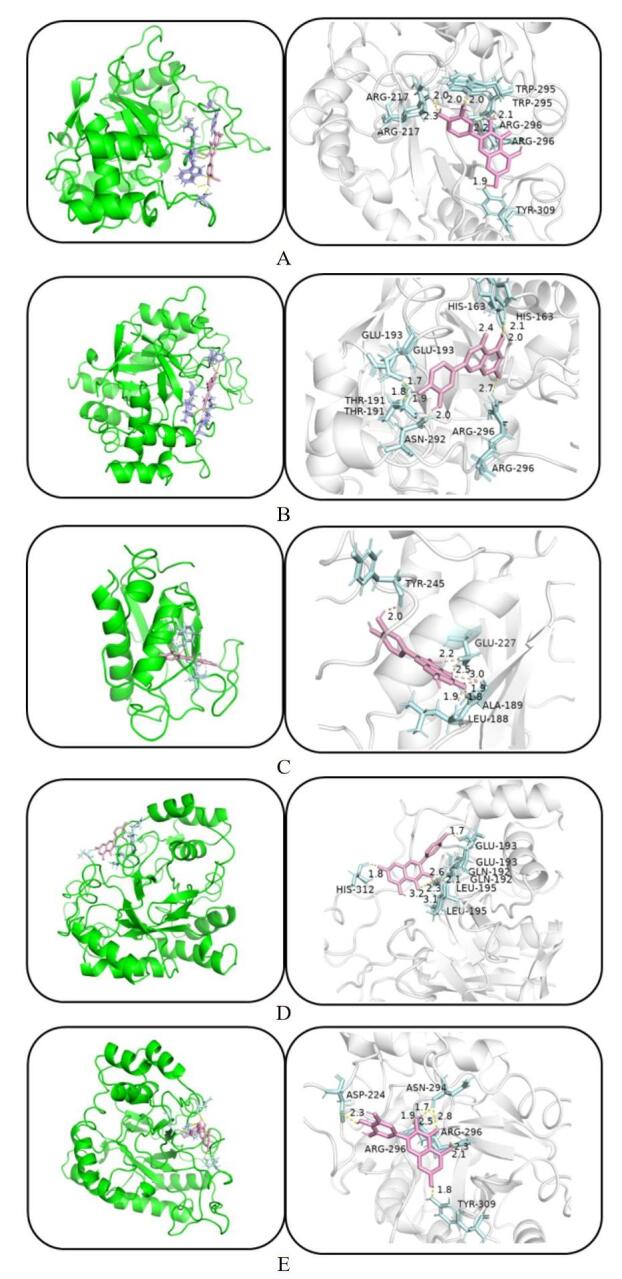
各关键成分与核心靶点蛋白对接详情模拟

### 2.3 动物实验验证

#### 2.3.1 各组小鼠背部急性湿疹皮损程度评分及搔抓次数测定

如图14所示，采取EASI评分法观察各组小鼠，如表6所示，与空白组相比，其余组别在DNCB溶液造模后，小鼠的皮肤评分明显升高。造模期间小鼠在DNCB给药后可明显观察到抓挠背部，背部出现明显红斑和抓痕，造模结束后给药治疗过程中，小鼠的症状改善，湿疹皮损程度评分降低。与模型组相比，川西合耳菊治疗高、中、低剂量组与阳性组的小鼠背部评分症状均有好转（*P<*0.01），说明经川西合耳菊和醋酸地塞米松的预防给药后，小鼠的急性炎症反应受到明显抑制。从表6中的Skin lesion scores和Scratching frequency的数值可以看出，阳性组与川西合耳菊水煎液高剂量组相比，醋酸地塞米松组的数据更小，表明醋酸地塞米松对小鼠的急性湿疹炎症反应的治疗效果更好。

**图14:**
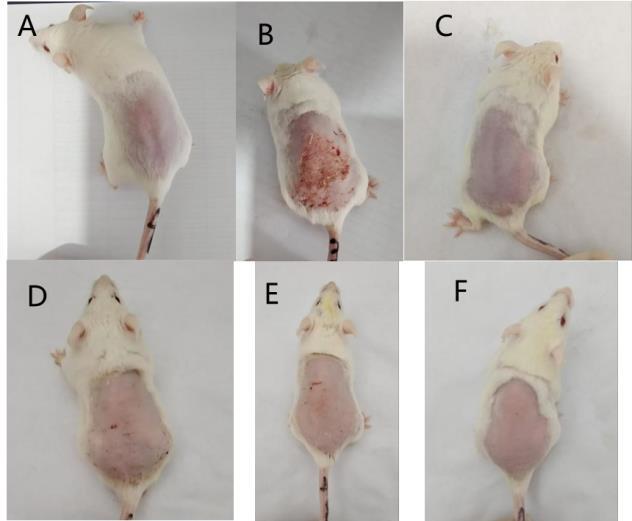
各组小鼠皮肤损伤图片

**表6:** 各组小鼠皮损评分和搔抓次数（*n*=10）

Group	Skin lesion scores	Scratching frequency
Blank group	0.50±0.31	2.67±2.08
Model group	3.87±0.21^**^	62.00±8.54^**^
Positive group	1.46±0.19^**##^	25.33±4.51^**##^
High-dose group	1.79±0.29^**##^	31.33±4.04^**##^
Middle-dose group	2.12±0.31^**##^	40.67±4.04^**##^
Low-dose group	2.83±0.20^**##^	45.00±3.00^**##^

Compared with the blank group， ** *P*<0.01； compared with the model group， ## *P*<0.01.

#### 2.3.2 各组小鼠脾脏、胸腺和肝脏指数的测定

如表7所示，与空白组相比，模型组和川西合耳菊低剂量治疗组小鼠的脾脏指数、肝脏指数和胸腺指数均升高（*P*<0.05）；与模型组相比，川西合耳菊高/中治疗组和阳性组小鼠的脾脏指数、肝脏指数和胸腺指数均降低（*P*<0.01）；与模型组相比，川西合耳菊低/高剂量治疗组均能有效恢复小鼠免疫平衡（*P*<0.05），与阳性组相比，差异无统计学意义（*P*>0.05），表明治疗效果与阳性药相当。

**表7:** 各组小鼠脾脏、胸腺和肝脏指数的测定（*n*=10）

Group	Spleen index	Liver index	Thymus index
Blank group	23.58±1.19	329.90±8.88	17.52±0.99
Model group	45.89±4.97^**^	395.29±26.37^**^	29.55±3.66^**^
Positive group	25.92±1.87^##^	326.58±12.59^##^	18.30±4.89^##^
High-dose group	25.81±1.43^##^	339.62±17.31^##^	20.39±0.38^##^
Middle-dose group	27.63±0.38^##^	355.85±1.65^##^	22.01±2.27^##^
Low-dose group	34.74±4.99^**##^	375.44±33.93^**#^	22.57±1.69^*##^

Compared with the blank group， * *P*<0.05， ** *P*<0.01； compared with the model group， # *P*<0.05， ## *P*<0.01.

#### 2.3.3 各组小鼠皮肤组织HE染色病理学检测

如图15所示，空白组小鼠皮肤表皮薄，真皮层皮肤分布规则，附属物、脂肪组织正常，无明显炎症浸润。模型组表皮角化过度，增厚明显，棘层增厚，表皮内细胞间水肿，皮肤组织致密，炎性细胞于皮下浸润。川西合耳菊治疗组高、中、低剂量组及阳性组与模型组相比较均有所改善，阳性组的表皮较薄，无明显炎症浸润，而川西合耳菊治疗组低、中、高剂量组可见表皮厚度依次降低，炎症细胞浸润现象也依次改善，其中阳性对照组、川西合耳菊高剂量治疗组较模型对照组改善明显。

**图15:**
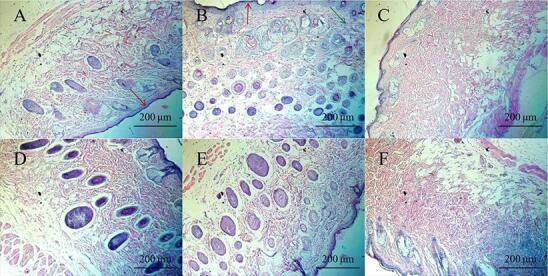
各组小鼠背部皮肤组织HE染色结果图（200×）

#### 2.3.4 各组小鼠血清中IFN-γ、TNF-α、IL-6、IL-17的含量比较

如表8所示，与空白组相比，模型组小鼠血清中TNF-α、IL-6、IL-17含量均显著升高（*P*<0.01）；与模型组相比，阳性组、川西合耳菊高中剂量组小鼠血清中TNF-α、IL-6、IL-17含量均显著降低（*P*<0.05），川西合耳菊低剂量组小鼠血清中的TNF-α与IL-17含量显著降低（*P*<0.05），而川西合耳菊低剂量组小鼠血清中IL-6含量与模型组相比无统计学意义（*P*>0.05）。与空白组相比，模型组小鼠血清中IFN-γ含量均显著降低（*P*<0.01）；与模型组相比，阳性组、川西合耳菊高中剂量组小鼠血清中IFN-γ含量均显著升高（*P*<0.05），而川西合耳菊低剂量组小鼠血清中IFN-γ含量与模型组相比无统计学意义（*P*>0.05）。

**表8:** 各组小鼠血清中TNF-α、IFN-γ、IL-6、IL-17的蛋白水平 (pg/mL)

Group	IFN-γ	TNF-α	IL-6	IL-17
Blank group	628.89±75.43	202.17±36.18	216.10±69.31	21.98±7.60
Model group	433.25±20.38^**^	395.84±45.93^**^	405.90±50.68^**^	72.21±13.32^**^
Positive group	604.92±69.82^##^	245.46±46.31^##^	260.20±44.66^##^	37.79±3.80^**##^
High-dose group	600.66±34.15^##^	267.74±18.18^**##^	304.62±72.74^##^	43.97±6.85^**##^
Middle-dose group	519.82±52.74^*#^	292.08±43.27^**##^	311.29±58.82^*#^	46.93±11.67^**##^
Low-dose group	498.66±76.46^**^	347.84±22.91^**#^	332.93±60.88^**^	57.97±11.87^**#^

Compared with the blank group， * *P*<0.05， ** *P*<0.01； compared with the model group， # *P*<0.05， ## *P*<0.01.

湿疹的发病机制可能与T细胞亚群（Th1/Th2）平衡失调有关^［73］^，在生理状态下，Th1细胞通过分泌IFN-γ、TNF-α等I型细胞因子激活巨噬细胞介导的细胞免疫应答，而Th2细胞则通过IL-4、IL-5、IL-13等Ⅱ型细胞因子驱动体液免疫反应。TNF-α具有介导炎症、组织损伤、休克、调节机体的免疫功能等病理生理反应；IFN-γ在抗感染、抗肿瘤及免疫调节中发挥关键作用，并且其有促炎和免疫保护双重效应；IL-17是Th17细胞分泌的特征性细胞因子，IL-17的生物活性有参与炎症级联放大，自身免疫调控，感染免疫防御及屏障功能破坏的病程等^［74］^。IL-6具有促炎和抗炎两种作用，其可介导MAPK通路、PI3K/Akt通路。在急性湿疹模型中，机体呈现Th2免疫优势，Th2过度活化可促进IgE大量产生并抑制Th1免疫应答，导致模型组IFN-γ水平降低；药物干预后IFN-γ显著回升，提示药物可恢复Th1免疫功能，通过增强Th1应答抑制过度的Th2型过敏反应与IgE异常升高，进而纠正Th1/Th2免疫失衡，发挥治疗急性湿疹的作用。

#### 2.3.5 各组小鼠皮肤组织中PI3K、Akt、TNF-α、IL-6 mRNA相对表达水平比较

如表9所示，与空白组相比，模型组小鼠皮肤组织中PI3K、Akt、TNF-α、IL-6 mRNA表达量均有显著升高，差异有统计学意义（*P*<0.01）；与模型组相比，阳性组、川西合耳菊高/中剂量组小鼠皮肤组织中PI3K、Akt、TNF-α、IL-6 mRNA表达量均有所下降（*P*<0.01），川西合耳菊低剂量组小鼠皮肤中的PI3K mRNA表达量与模型组相比，具有显著性差异（*P*<0.05），而Akt、TNF-α、IL-6 mRNA表达量与模型组相比无显著性差异（*P*>0.05）。

**表9:** 各组小鼠皮肤组织中PI3K、Akt、TNF-α及IL-6 mRNA相对表达水平

Group	PI3K	Akt	TNF	IL-6
Blank group	1.07±0.07	0.92±0.08	1.04±0.07	0.94±0.05
Model group	3.02±0.16^**^	2.08±0.08^**^	2.18±0.12^**^	1.78±0.03^**^
Positive group	1.15±0.12^##^	1.08±0.08^##^	1.10±0.10^##^	1.03±0.03^##^
High-dose group	1.76±0.10^**##^	1.35±0.16^**##^	1.42±0.43^**##^	1.23±0.05^**##^
Middle-dose group	2.18±0.05^**##^	1.70±0.07^**##^	1.73±0.07^**##^	1.45±0.09^**##^
Low-dose group	2.71±0.27^**#^	1.99±0.08^**^	2.07±0.16^**^	1.70±0.12^**^

Compared with the blank group， * *P*<0.05， ** *P*<0.01； compared with the model group， # *P*<0.05， ## *P*<0.01.

#### 2.3.6 各组小鼠皮肤组织中PI3K、p-PI3K、Akt、p-Akt蛋白表达水平比较

由图16和表10可知，与空白组相比，模型组中小鼠皮肤组织中p-PI3K/PI3K、p-Akt/Akt表达水平显著升高，差异具有统计学意义（*P*<0.01）；与模型组相比，阳性组、川西合耳菊高/中剂量组中小鼠皮肤组织中p-PI3K/PI3K、p-Akt/Akt表达水平均降低，差异具有统计学意义（*P*<0.01）；川西合耳菊低剂量组中小鼠皮肤组织中p-PI3K/PI3K、p-Akt/Akt表达水平 与模型组相比无显著性差异（*P*>0.05）。

**图16:**
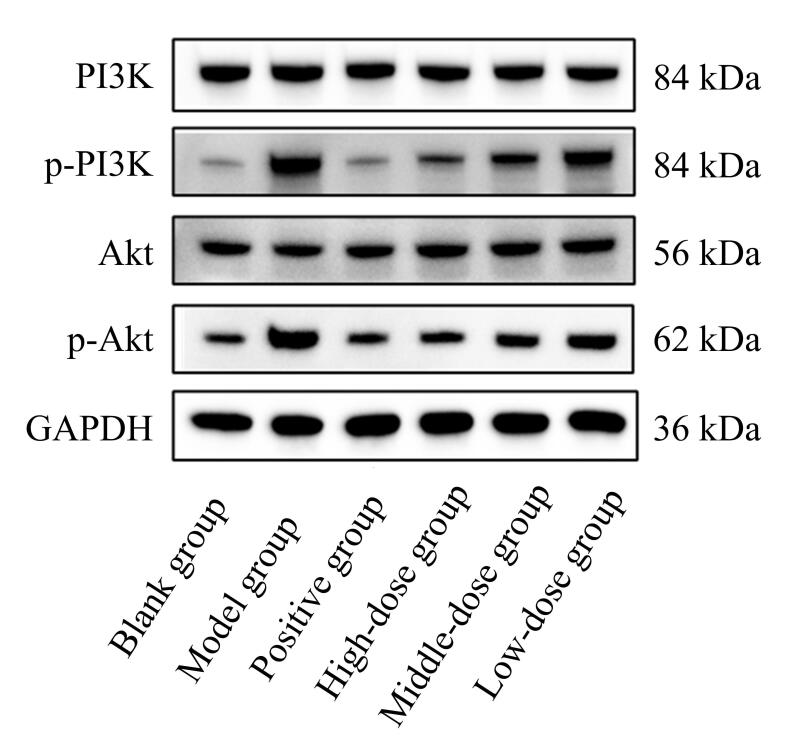
各组小鼠皮肤组织PI3K、p-PI3K、Akt、p-Akt蛋白表达

**表10:** 各组小鼠皮肤组织中PI3K、p-PI3K、Akt及p-Akt蛋白相对表达水平比较（*n*=6）

Group	p-PI3K/PI3K	p-Akt/Akt
Blank group	1.00±0.13	0.99±0.13
Model group	2.57±0.22^**^	2.27±0.24^**^
Positive group	1.23±0.11^##^	1.11±0.16^##^
High-dose group	1.60±0.12^**##^	1.56±0.03^**##^
Middle-dose group	1.97±0.17^**##^	1.93±0.06^**##^
Low-dose group	2.51±0.08^**^	2.25±0.04^**^

Compared with the blank group， ** *P*<0.01； compared with the model group， ## *P*<0.01.

## 3 结论

本研究从“成分鉴定-靶点预测-机制解析”多维度解析川西合耳菊治疗急性湿疹的科学内涵，通过网络药理学及体内动物实验验证，证明川西合耳菊通过“多成分-多靶点-多通路”协同机制治疗急性湿疹，可能通过降低TNF-α、IL-6、IL-17含量，升高IFN-γ的含量来恢复Th1/Th2平衡；通过抑制PI3K/Akt信号通路来减轻炎症反应，改善急性湿疹小鼠的临床症状，阐明其通过多靶点调控炎症信号网络及免疫平衡的作用模式。因此，后续的机制探索应紧密围绕网络药理学富集结果PI3K-Akt、MAPK等信号通路展开，精准挖掘川西合耳菊在这些通路中的关键作用节点，力求全面揭示川西合耳菊抗急性湿疹的深层机制。
